# Changes in the Porcine Intestinal Microbiome in Response to Infection with *Salmonella enterica* and *Lawsonia intracellularis*

**DOI:** 10.1371/journal.pone.0139106

**Published:** 2015-10-13

**Authors:** Klaudyna A. Borewicz, Hyeun Bum Kim, Randall S. Singer, Connie J. Gebhart, Srinand Sreevatsan, Timothy Johnson, Richard E. Isaacson

**Affiliations:** Department of Veterinary and Biomedical Sciences, University of Minnesota, 1971 Commonwealth Ave., St. Paul, MN, 55108, United States of America; University of Saskatchewan, CANADA

## Abstract

*Salmonella enterica* is a leading cause of food borne illness. Recent studies have shown that *S*. *enterica* is a pathogen capable of causing alterations to the composition of the intestinal microbiome. A recent prospective study of French pork production farms found a statistically significant association between *Lawsonia intracellularis* and carriage of *S*. *enterica*. In the current study the composition of the gut microbiome was determined in pigs challenged with *S*. *enterica* serovar Typhimurium and or *L*. *intracellularis* and compared to non-challenged control pigs. Principal coordinate analysis demonstrated that there was a disruption in the composition of the gut microbiome in the colon and cecum of pigs challenged with either pathogen. The compositions of the microbiomes of challenged pigs were similar to each other but differed from the non-challenged controls. There also were statistically significant increases in *Anaerobacter*, *Barnesiella*, *Pediococcus*, *Sporacetigenium*, *Turicibacter*, *Catenibacterium*, *Prevotella*, *Pseudobutyrivibrio*, and *Xylanibacter* in the challenged pigs. To determine if these changes were specific to experimentally challenged pigs, we determined the compositions of the fecal microbiomes of naturally infected pigs that were carriers of *S*. *enterica*. Pigs that were frequent shedders of *S*. *enterica* were shown to have similar fecal microbiomes compared to non-shedders or pigs that shed *S*. *enterica* infrequently. In a comparison of the differentially abundant bacteria in the naturally infected pigs compared to experimentally challenged pigs, 9 genera were differentially abundant and each exhibited the same increase or decrease in abundance between the two groups. Thus, there were similar changes in the GI microbiome associated with carriage of *S*. *enterica* regardless of whether the pigs were experimentally challenged with *S*. *enterica* or acquired it naturally.

## Introduction

*Salmonella enterica* is one of the most common causes of food borne diarrheal disease. The Centers for Disease Control has estimated that *S*. *enterica* is responsible for over a 1.5 million cases of food borne illness per year in the United States [1]. *S*. *enterica* has been ranked as the leading cause of bacterial food borne disease as measured by the combined cost of illness and Quality Adjusted Life-Year [2]. It has been estimated that the economic losses due to salmonellosis in the US exceed $3.5 billion per year. Pigs are known to be an important source of *S*. *enterica* and are frequent asymptomatic carriers of *S*. *enterica*. *S*. *enterica* is believed to enter the food chain by establishing persistent but asymptomatic infections of pigs. Persistent infections can be experimentally established early in life and once established *S*. *enterica* can persist for the life of the animal [3–5]. Persistently infected pigs intermittently shed *S*. *enterica* in feces but usually in low numbers (10–100 cells per gram of feces) [5]. Even though pigs show no signs of infection or disease they may sporadically shed the organism in feces. Stresses, including transport and feed withdrawal, promote the resumption of fecal shedding just prior to slaughter, which increases the risk of contaminating meats at the time of processing, and may be responsible for spread to uninfected animals held in lairage [5,6]. Thus, swine can act as a reservoir for the spread of *S*. *enterica* throughout the herd, within the packing plant, and during processing to finished product [5,7–9].

There is a growing body of evidence that suggests that interactions between members of the gut microflora (microbiome) of livestock animals contribute to the health and well being of those animals [10,11]. For example, studies employing experimental challenges of mice and chickens with *S*. *enterica* have shown that significant alterations of the gastrointestinal (GI) microbiome occur after challenges [12,13]. In one study, Stecher *et*. *al*. also showed a link between inflammation caused by *S*. *enterica* and alterations of the colonic microbiome, providing *S*. *enterica* with a competitive advantage to colonize the microbe rich colon [14].

While a comprehensive description of the pig gut microbiome is just now being accumulated, it has been proposed that microbes in the GI tract form networks of symbiotic relationships that result in exclusion of pathogens. Changes in the structure and function of the microbial ecosystem can lead to microbial dysbiosis and predispose the host to colonization with pathogens. (Dysbiosis is defined as an imbalance in the microbial composition in a specific habitat). It is likely that infection with one pathogen can promote further infections with other pathogens. For example, recent work by Beloeil *et al* [15] proposed that infection of pigs with the enteric pathogen *Lawsonia intracellularis* might predispose them to shed *S*. *enterica*. In their study of 105 French pork production farms they found a statistically significant association (Odds Ratio 3.2, 90% Confidence Interval 1.4 to 7.2) between infections with *L*. *intracellularis* and carriage of *S*. *enterica*. This observation is consistent with the hypothesis that *L*. *intracellularis* interacts with *S*. *enterica* and/or other members of the gut microbiome and that these interactions lead to increased colonization and shedding of *S*. *enterica*. Since *L*. *intracellularis*, the cause of porcine proliferative enteropathy, colonizes the ileum and *S*. *enterica* colonizes the colon and cecum, we assume that *L*. *intracellularis* and *S*. *enterica* likely have indirect interactions that might be mediated by other members of the GI microbiome.

The work reported here was performed to determine if infection with *S*. *enterica* and *L*. *intracellularis* individually or together was capable of causing a dysbiosis of the pig gut microbiome. To do this we experimentally challenged pigs with *S*. *enterica* and/or *L*. *intracellularis* and measured the composition of the gut microbiome at four sites in the GI tract and compared the compositions to non-infected pigs. To determine if the changes in the microbiome were applicable to normal swine rearing practices where natural infections are common but mainly result in low level shedding of *S*. *enterica*, we also looked for microbiome changes in the GI tracts of commercial pigs that were naturally infected with *S*. *enterica* and compared the observed compositional changes to the changes in the experimentally infected pigs. We found that experimental infections of pigs *L*. *intracellularis*, *S*. Typhimurium, or both lead to changes in the composition of the microbiome in the intestinal tract. Pigs naturally infected with *S*. *enterica* have similar changes in their fecal microbiomes.

## Materials and Methods

### Animals and experimental design

The animal protocol used was approved by the University of Minnesota Institutional Care and Uses Committee protocol #0912A75576. For experimental challenges a total of 28 4-week old crossbred, weaned pigs were obtained and randomly allocated into four pens (7 pigs per group). Empty pens were between each of the pens used for the pigs so that the pigs in a group did not have direct contact with pigs in another group. At five weeks of age, four pigs (1 per pen) were removed and necropsied. Ten-centimeter segments of jejunum, ileum (approximately 10 centimeters from the ileocecal junction), cecum and ascending colon were removed. The remaining pigs were treated as follows: pigs in pen numbers three and four were challenged orally with 1 x 10^10^ CFU *L*. *intracellularis* strain PHE/MN1-00. One week later (when the pigs were six weeks of age) pigs in pen numbers 2 and 4 were challenged orally with 2 x 10^8^ CFU of nalidixic acid resistant *S*. Typhimurium strain 798. Pigs in pen number 1 remained unchallenged and served as controls. When the pigs were 7, 9, and 11 weeks of age two pigs from each pen were euthanatized with an overdose of sodium pentobarbital and necropsied.

For the field-based animal protocol commercial pigs were utilized. Pigs were from a commercial pig farm located in southwestern Minnesota, U.S.A (43°37’26”N95°35’57”W). These pigs were part of a larger study performed to describe microbiome changes in pigs fed the antimicrobial growth promoter tylosin [16]. The production barn contained 20 pens and each pen had 25 pigs. A single pen in the middle of the barn was selected for sampling. Pigs were kept in the same pen for the entire sampling period without introduction of any new pigs. Ten pigs from the total of 25 were randomly selected for sampling and ear tagged for identification. Fresh fecal samples from each of the ear tagged animals were individually collected from the pigs’ rectums. Samples were collected five times over their growth period at 3-week intervals starting when the pigs were 10-weeks old. Pigs did not receive antibiotics in feed or for any therapeutic purposes prior to and during the sampling period.

### Growth of bacterial challenge inocula

The nalidixic acid resistant *S*. *enterica* serovar Typhimurium strain 798 was prepared by overnight growth in LB broth. *L*. *intracellularis* strain PHE/MN1-00 was grown in McCoy cells as previously described [17].

### Tissue sampling, DNA extraction from tissues and feces, and DNA sequencing

The 10 cm of cecum and colon samples were opened longitudinally and the mucosal layer scraped with a microscope slide to remove loosely associated contents and most of the mucosal tissue. The fibrous serosal tissue was discarded. The mucosal samples were weighed and 1 gram of each was used to determine the concentrations of *S*. Typhimurium as described below. DNA was extracted from a second 1 gram sample as previously described [18]. The DNA extraction protocol employed two rounds of bead beating in the presence of NaCl and sodium dodecyl sulfate, followed by sequential ammonium acetate and isopropanol precipitations. The precipitated nucleic acids were then treated with RNase A and proteinase K, and the DNA purified using a QIAgen DNA Mini Stool Kit (Valencia, CA), according to manufacturer's recommendations. For fecal samples, DNA was extracted from the 1 gram of feces using the same extraction protocol.

### Detection of *S*. *enterica* and *L*. *intracellularis*

*S*. *enterica* in tissue and fecal samples was detected as previously described [19]. One gram of tissue was suspended in 9 ml tetrathionate broth (TTB) and incubated at 41°C for 24 hours. One hundred μl was then transferred to 900 μl Rappaport-Vassiliadis R10 broth (Remel, Lenexa, KS) and incubated for 24 hours at 41°C. Growth from the Rappaport-Vassiliadis R10 broth was plated on XLT4 agar plates (Becton Dickinson, Baltimore, MD) and incubated at 37°C for 24 hours. Suspect *S*. *enterica* colonies were confirmed as *S*. *enterica* using API^®^20E strips (BioMerieux^®^ SA, Marcy-I’Etoiele, France) and by PCR using primers specific for the gene *invA* (Forward: *invA* ACAGTGCTCGTTTACGACCTGAAT and Reverse: *invA* AGACGACTGGTACTGATCGATAAT) [20]. For detection of *S*. *enterica* serovar Typhimurium strain 798, colonies from the XLT-4 plates were re-plated on LB agar containing nalidixic acid (100 μg/ml). Template DNA was prepared from *S*. *enterica* isolates using a boiling lysis procedure [21]. The PCR mixtures contained 12.5 μl of Taq polymerase (Promega, Madison, WI), 0.2 μM of the primer pairs, and 2 μl of DNA template in a 25μl reaction. Cycling conditions started with an initial denaturation at 95°C for 2 minutes followed by 30 cycles at 95°C for 30 seconds, 60°C for 30 seconds, and 72°C for 1 minute. A final 7 minute extension was incubated at 72°C.

*S*. *enterica* isolates were serotyped by the National Veterinary Services Laboratory (Ames, Iowa).

Detection and quantification of *L*. *intracellularis* was performed using a quantitative PCR protocol previously described [17]. The qPCR assay is based on the detection of the aspartate ammonia-lyase (*aspA*) gene.

### Analysis of the intestinal microbiomes of intestinal tissues

PCR primers that flanked the V3 hypervariable region of bacterial 16S rRNAs were designed based on the blueprint for barcodes [18,22]. The oligonucleotide primers included Roche A or B sequencing adapters at the 5' ends and template specific sequences at the 3' end. Barcodes were located in between the sequencing adapter and the template specific sequences of the forward primer. The primer sequences were: 5' (sequence adapter)– 10-base barcode—CCTACGGGAGGCAGCAG 3' (forward) and 5' (sequence adapter)–ATTACCGCGGCTGCTGG 3' (reverse) [22,23]. The amplification mix contained 2.5 units of Taq polymerase, 0.2mM of dNTPs, 0.4μM of each fusion primer, and 50ng of DNA in a reaction volume of 50μl. Cycling conditions started with an initial denaturation at 95°C for 2 minutes followed by 20 cycles at 95°C for 30 seconds, 60°C for 30 seconds, and 72°C for 30 seconds. A final 7 minute extension was incubated at 72°C. For the jejunal and ileal samples, the number of PCR cycles was increased to 25 to compensate for lower bacterial numbers in these samples compared to the cecal and colonic samples. The PCR amplicon products were separated on a 1.5% agarose gels, extracted from the gels and then were cleaned using QIAgen DNA Mini Stool Kits (Valencia, CA). The quality of the product was assessed on a Bioanalyzer 2100 (Agilent, California, USA). Only PCR products without primer dimers and contaminant bands were used for sequencing. The PCR amplicons were sequenced using a Roche 454 GS-FLX sequencer (454 Life Sciences, Branford, CT).

To minimize effects of random sequencing errors, we eliminated (*i*) sequences that did not appropriately match the PCR primer and the barcode at the beginning of a read, (*ii*) sequence reads with <50 bases after the proximal PCR primer if they terminated before reaching the distal primer, and (*iii*) sequences that contained more than one undetermined nucleotide (N). Mothur implemented in Galaxy was used for quality assessments including sequence trimming and assignment of operational taxonomic units using a 97% similarity cutoff [24]. Chimeras were removed using Chimera Slayer implemented in UCHIME [25]. A phylogenetic assessment was conducted using RDP classifier with a bootstrap cutoff of 50%. DNA sequence data is freely available the through the University of Minnesota data conservancy: http://conservancy.umn.edu/handle/11299/172268?show=full.

### Statistical analysis

Determination of statistically unique taxa was determined using MetaStats [18]. MetaStats is a tool that was developed to identify differentially abundant species among samples. It uses the nonparametric t-test, Fisher’s exact test, and the false discovery rate to identify prioritized lists of relevant features. Principal coordinate analysis (PCoA) plots were generated by using weighted Fast UniFrac [26].

## Results

### Microbiome composition in different GI sites of experimentally infected pigs

**Fig 1:**
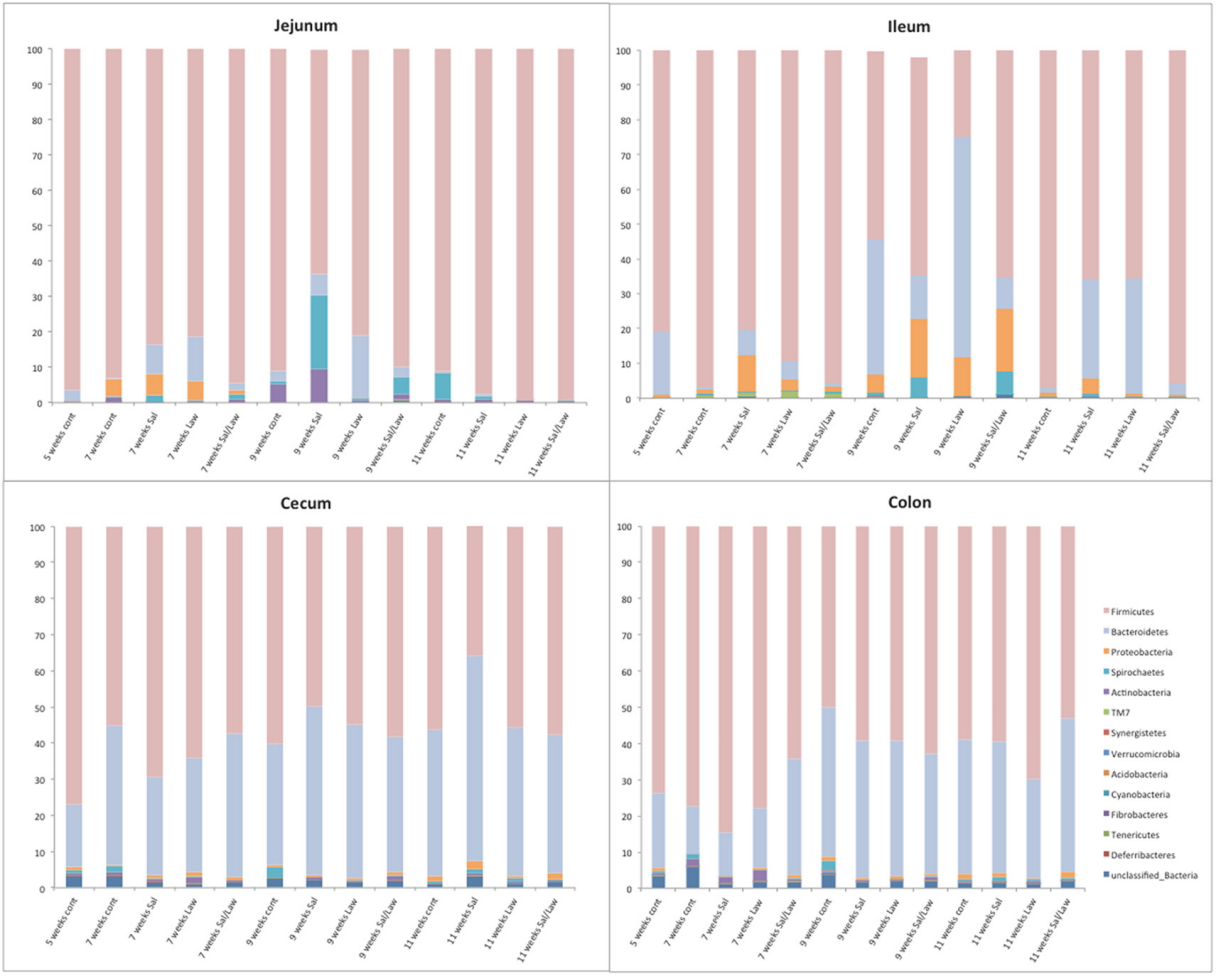
Distribution of bacterial phyla in pig tissues. Graph of the distribution of different phyla in four pig tissues (jejunum, ileum, cecum, and colon) at different times post challenge with *S*. *enterica* serovar Typhimurium strain 798 (Sal), *Lawsonia intracellularis* strain PHE/MN1-00 (Law), or both (Sal/Law) as well as non-challenged controls (cont).

**Fig 2:**
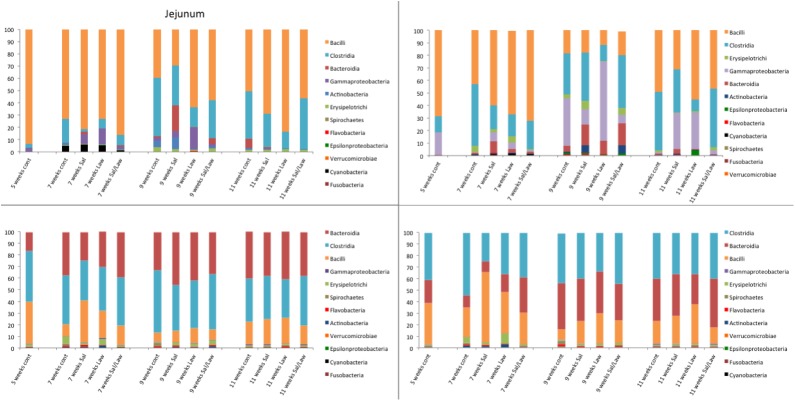
Distribution of bacterial classes in pig tissues. Graph of the distribution of different classes in four pig tissues (jejunum, ileum, cecum, and colon) at different times post challenge with *S*. *enterica* serovar Typhimurium strain 798 (Sal), *Lawsonia intracellularis* strain PHE/MN1-00 (Law), or both (Sal/Law) as well as non-challenged controls (cont).

The microbiomes in pigs experimentally challenged with *S*. *enterica* serovar Typhimurium (*S*. Typhimurium), *L*. *intracellularis*, or both were determined. *S*. Typhimurium is known to cause sub-clinical infections with sporadic low level shedding in feces, while *L*. *intracellularis* is a pig pathogen that causes proliferative enteropathy in the lower small intestines. To test this hypothesis, groups of pigs were experimentally challenged orally with one or both microbes (*S*. Typhimurium strain 798 and/or *L*. *intracellularis* strain PHE/MN1-00). At multiple times pigs were euthanized and tissue samples collected. Total DNA was extracted from the jejunum, ileum, cecum, and colon of these pigs and the extracted community DNA was PCR amplified and sequenced using primers specific for the V3 region of the *16S rRNA* gene. On average, 23,160 DNA sequences were obtained per sample. Taxonomic assignments were made using RDP Classifier and are shown in Fig 1 (phylum) and Fig 2 (class). The taxonomic assignments are shown in S1 Table. The two most common phyla in all samples at all times were Firmicutes followed by Bacteroidetes. These two phyla dominated the cecum and colon samples accounting for greater than 95% of all bacteria. In the cecum and colon tissue samples, Firmicutes were the most common bacteria ranging from 65.4% to 85% in seven-week old pigs, 55.3% to 63.1% in the nine-week old pigs, and 56.4% to 70.7% in the eleven-week old pigs. In the jejunum, Firmicutes and Bacteroidetes were the most predominant phyla observed (greater than 95%). In the ileal samples, Firmicutes was the most predominant phylum (64% to 96% in the seven-week old pigs, 25% to 65.9% in the nine-week old pigs, and 65.6% to 85% in the eleven-week old pigs. They also contained higher levels of Proteobacteria. These results are similar to those obtained by Looft, *et*. *al*. who found a similar composition in the ileum of pigs [27].

At the class level, Bacilli and Clostridia were dominant in the jejunum and ileum, but depending upon the time of sampling and treatment, Gammaproteobacteria and Bacteroidia were the most dominant classes. In the cecum and colon, Bacteroidia, Clostridia, and Bacilli dominated representing greater than 90% of all bacteria. A summary of the entire taxonomic classification data is available in S2 Table.

Using culture based methods, *S*. Typhimurium strain 798 was detected in all tissue samples from pigs challenged with *S*. *enterica* serovar Typhimurium strain 798 at 7, 9, and 11 weeks of age. The *S*. Typhimurium challenge strain was not detected prior to challenge (5 week old pigs) or in non-challenged pigs. *L*. *intracellularis* was found in samples from pigs challenged with *L*. *intracellularis* strain PHE/MN1-00 that were 9 or 11 weeks of age but not in pigs that were 7 weeks of age or that were not challenged. At 9 weeks of age, *L*. *intracellularis* was found in the jejunum, ileum, cecum, and colon samples and at 11 weeks of age it was only found in the cecum and colon samples.

### Principal coordinate analysis of all pig tissues

**Fig 3:**
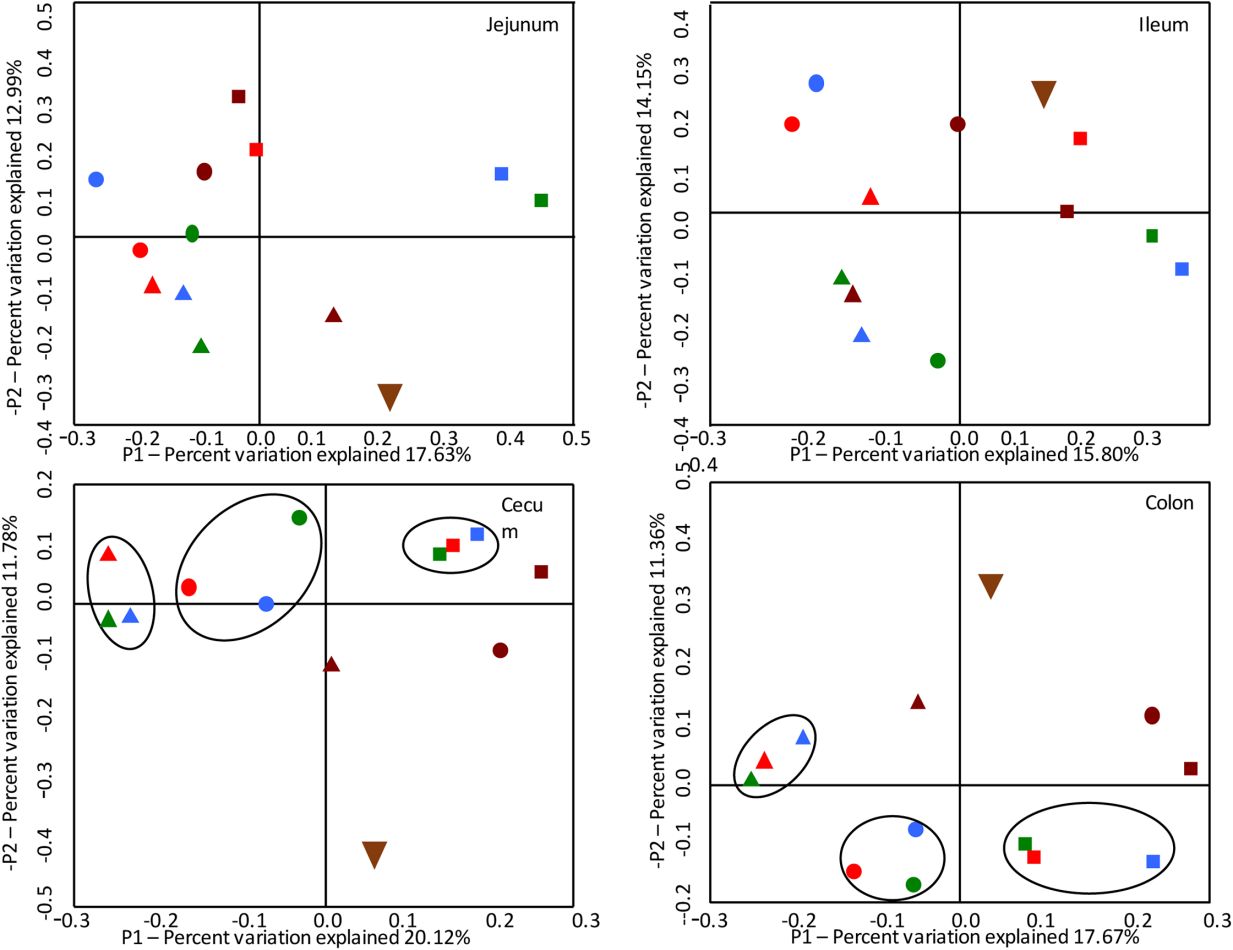
Principal coordinate analysis graphs comparing the total compositions of the microbiomes in four pig tissues (jejunum, ileum, cecum, and colon) at various times. The symbol shape associated with each data point represents the time of sample collection (5 (upside down triangle), 7 (triangles), 9 (circles), and 11 (squares) weeks of age). The symbol color reflects the treatment: maroon = non-challenged controls, red = challenged with *S*. *enterica* serovar Typhimurium strain 798, green = *Lawsonia intracellularis* strain PHE/MN1-00, blue = both challenge pathogens.

Fast UniFrac [26] was used to perform principal coordinate analysis to compare the overall compositions of the microbiomes in the various treatment groups over time (Fig 3). While the compositions of the microbiomes in the jejunum and ileum did not segregate into distinct sector of the plot based on treatment, segregation was observed in comparing microbiome compositions in the cecum and colon. The group compositions of challenged pigs, regardless of the challenge group, were similar to each other at each time point, but differed from the non-challenged controls. Also, the overall compositions of the microbiome in all challenge groups differed from each other by time rather than treatment.

### Microbiome composition differences in the four GI tissues

**Fig 4:**
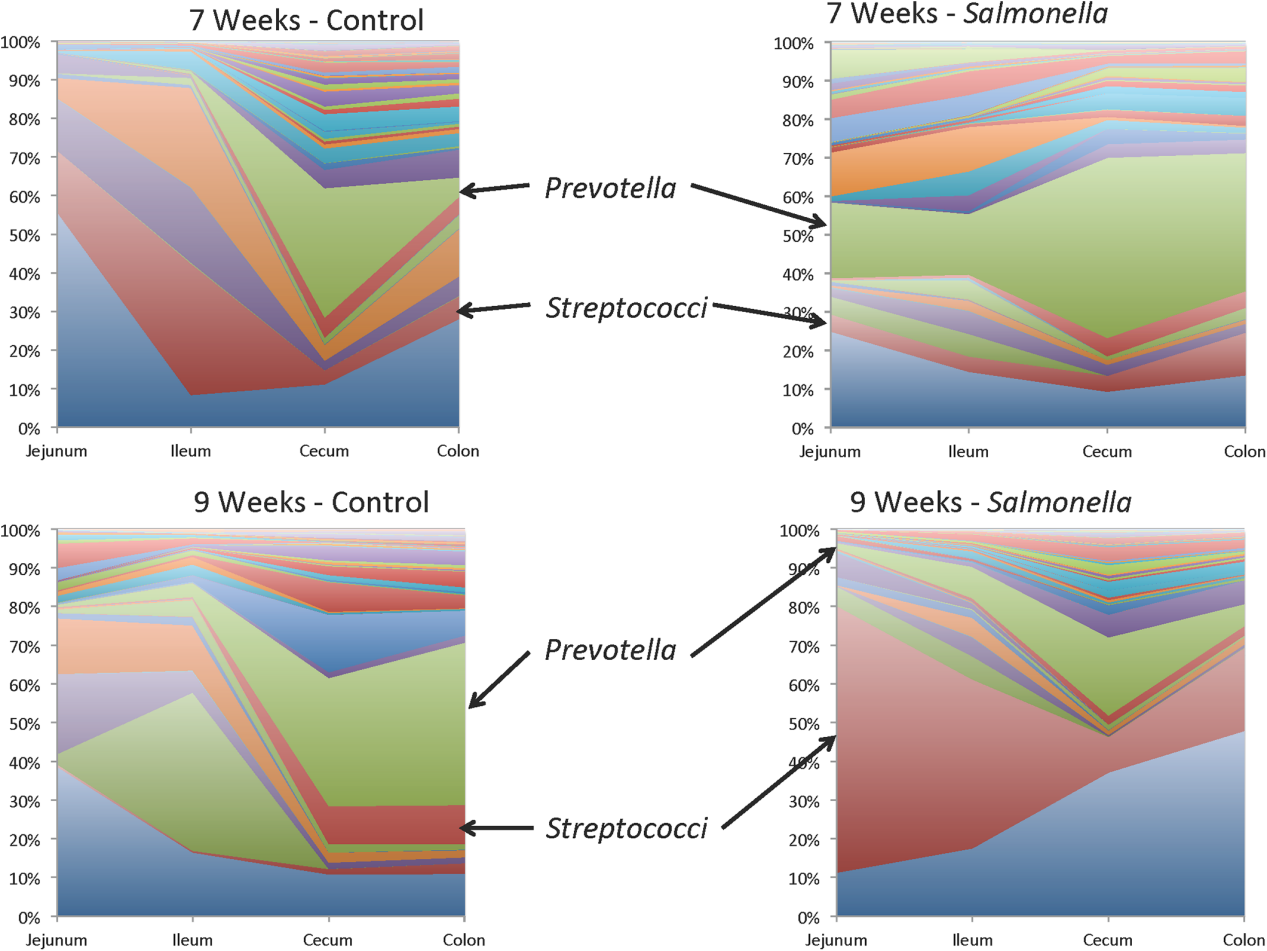
Comparison (mean of the group) of the tissue (jejunum, ileum, cecum, and colon) microbiomes of pigs at 7 or 9 weeks of age that were not challenged (control) or were challenged with *S*. *enterica* serovar Typhimurium strain 798 (Salmonella). The colors represent the same genus in all four panels. A figure legend can be found in S1 Table.

To compare the tissue-specific microbiomes of pigs in the four experimental groups, we plotted the relative concentration of the genus level assignments as the mean of the challenge group (S1, S2, S3 and S4 Figs) at each of the times sampled. Many differences can be seen and one example is shown in Fig 4 where the microbiomes of control and *S*. Typhimurium challenged pigs at 7 and 9 weeks of age are shown. Compared to control samples, there was an increase in the percentage of *Prevotella* present in the jejunal and ileal tissue samples at 7 weeks of age (1 week after challenge with *S*. *enterica*). By nine weeks of age the levels of *Prevotella* decreased but there was an increase in the level of *Streptococci* in the jejunal and ileal tissues. Looking at the non-challenged controls, the compositions of the microbiomes in each tissue within the same pig were shown to vary by tissue (site specific colonization) and time of sampling (microbial succession).

### Differentially abundant bacteria in the colon of experimentally infected pigs

To identify phyla that were differentially abundant, MetaStats [18] was used with a probability cut off of P< 0.05. Because cecum and colon tissues yielded similar PCoA plots and because no association between treatment group and jejunum and ileum were observed, only the colon samples were included in this analysis. In the seven-week old pigs (2 weeks post challenge with *L*. *intracellularis* and 1 week post challenge with *S*. Typhimurium) the colon of the dual infected group contained fewer members of the phylum Bacteroidetes (Table 1). This difference was compared to the control and to the single pathogen infection groups. Pigs challenged with *L*. *intracellularis* had increased levels of Actinobacteria compared to the other groups. In pigs that were 9 weeks of age, there was a significant decrease in the number of Spirochaetes in all challenge groups compared to the control group. In the 11 week old pigs, members of the phylum Verrucomicrobia were decreased in each of the challenge groups although the decrease in the group challenged with *L*. *intracellularis* alone was not statistically significant. Members of the phylum Proteobacteria were reduced in the group challenged with *L*. *intracellularis* only.

**Table 1:** Distribution of phyla in the colon of pigs that are statistically unique (≤0.05)*.

	Phylum	Control	*Salmonella*	*Lawsonia*	*Salmonella/Lawsonia*
7 weeks	Bacteroidetes	79.79	85.01	78.78	65.38^a^
	Actinobacteria	1.89	1.49	3.13^c^	0.75
9 weeks	Spirochaetes	2.67	0^b^	0.33^b^	0.16^b^
	Fibrobacteres	0.19	0^b^	0.02	0^b^
11 weeks	Verrucomicrobia	0.18	0^b^	0.05	0^b^
	Proteobacteria	1.23	0.12	0.29^c^	0.91

* Numbers represent the percent of each phylum in the group

^a^ Compared to control, Salmonella, and Lawsonia

^b^ Compared to control

^c^ Compared to control, *Salmonella*, *and Salmonella/Lawsonia*

Differentially abundant bacteria at the genus level also were identified using MetaStats [28]. The results shown in S2 Table demonstrate numerous differences between the various groups. The differences are expressed as an overall increase or decrease in the relative abundance of the statistically unique genera compared to controls and the three treatment groups (*S*. Typhimurium, *L*. *intracellularis*, or *S*. Typhimurium and *L*. *intracellularis*) and then between each challenge group as well. Of particular interest was the consistent increase in *Anaerobacter* in seven-week old pigs receiving infectious challenges compared to the controls, increases in *Lactobacillus* in the pigs challenged with *L*. *intracellularis* and decreases in *Prevotella* in dual challenged pigs. In the nine-week old pigs, there were consistent increases in *Oribacterium* compared to control pigs. In the eleven-week old pigs, there was a consistent increase in *Paraprevotella* in pigs challenged with *L*. *intracellularis* and *S*. Typhimurium. These results demonstrate numerous perturbations of the colonic microbiome based on the challenge and time post challenge.

### Analysis of the fecal microbiome composition of commercial pigs that shed or did not shed *S*. *enterica*

**Fig 5:**
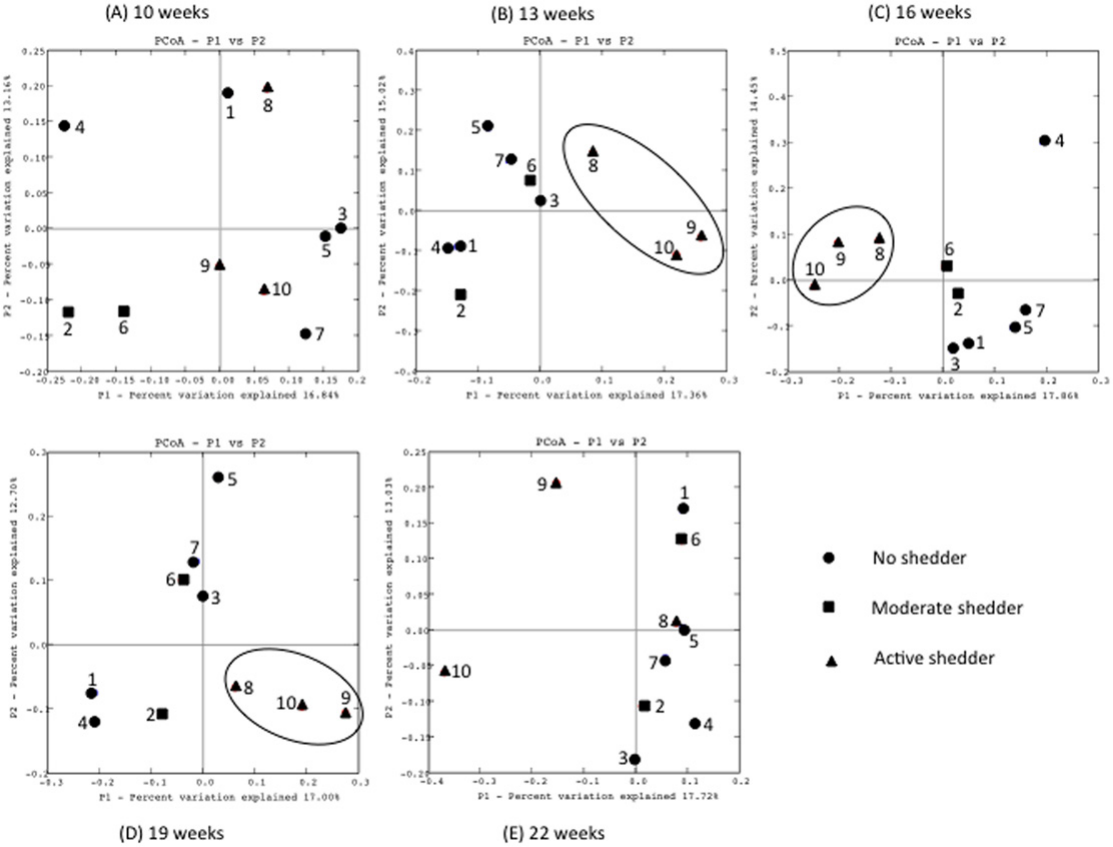
Principal coordinate analysis graphs comparing the total compositions of the fecal microbiome in 10 pigs at five times (10, 13, 16, 19, and 22 weeks of age) in pigs that were shedding or not shedding *S*. *enterica*. ● = Non-shedding pigs, ■ = moderate shedding pigs, ▲ = active shedding pigs.

Three levels of *S*. *enterica* shedding were defined for the commercial, naturally infected pigs based on *S*. *enterica* isolation from feces and PCR: non shedder (pigs 1, 3, 4, 5, and 7 were negative by both isolation and *invA* PCR at all sampling time points), moderate shedder (pigs 2 and 6 were positive by both tests at no more than two sampling times), and active shedder (pigs 8, 9, and 10 were positive by both tests at more than 3 sampling times) (Table 2). Pig 10 was shedding *S*. *enterica* at all time points. All but one of the *S*. *enterica* isolates were identified as serovar Manhattan. Pig 9 also shed serovar Typhimurium at the 16-weeks of age sampling time. The overall phylogenetic community composition was compared between active, moderate and non-shedding pigs using Fast UniFrac distance metric. Overall, the active shedders did appear to have a unique fecal microbiome composition, which was different from the moderate shedders and non-shedder. However, the fecal microbiome compositions of pigs sampled at 10 weeks of age did not segregate by shedding group. At sampling times 13, 16, and 19 weeks of age, pigs in the active *S*. *enterica* shedding group shared a similar community membership compared to pigs in the moderate and no-shedders group (Fig 5). Pig 10 was shedding *S*. *enterica* during the entire sampling period and it had a different microbiome composition compared to the other pigs when they were 22 weeks of age. By 22 weeks of age all of the other pigs had stopped shedding *S*. *enterica* (Table 2).

**Table 2:** Salmonella shedding status evaluated by PCR and isolation.

			Weeks of age				
Pig ID	Shedding status	Serotype	10	13	16	19	22
1	No		-	-	-	-	-
2	Moderate	Manhattan	+	+	-	-	-
3	No		-	-	-	-	-
4	No		-	-	-	-	-
5	No		-	-	-	-	-
6	Moderate	Manhattan	+	-	-	-	-
7	No		-	-	-	-	-
8	Active	Manhattan	+	+	+	+	-
9	Active	Manhattan	+	+	* +	-	-
10	Active	Manhattan	+	+	+	+	+

* Salmonella typhimurium

Using MetaStats, differentially abundant bacteria present in pigs infected with *S*. *enterica* were identified by comparison to pigs that never shed *S*. *enterica*. The results are shown in S3 Table. The top 10 most abundant bacteria at the 10 week time point were used as a point of reference to compare to the other sampling times. Of the top 10, *Fastidiosipela* was present in reduced numbers in all five samples, while *Pseudobutyriovibrio* was present at higher levels. *Prevotella*, *Lactobacillus*, *Anaerobacter*, *Treponema*, and *Roseburia* were present at reduced levels at four of the five sampling times while *Barnsiella* was at higher levels. *Campylobacter* an important food borne pathogen was present at lower levels at three of the five sampling times in *S*. *enterica* shedding pigs.

## Discussion

The work presented here was initiated to determine if specific pathogens caused a dysbiosis in the composition of the gut microbiome of pigs. Two approaches were used in the work. The first was to experimentally challenge pigs with *S*. Typhimurium, an important food borne pathogen, and/or *L*. *intracellularis*, the cause of proliferative enteropathy in pigs and then measure microbiome compositions over time and within different sites in the pig intestinal tract. The second approach was to analyze fecal samples collected from commercial pigs that were naturally infected and shedding low levels *S*. *enterica* in their feces and to compare the fecal microbiomes to pigs that were not shedding *S*. *enterica*. Using both approaches, the results demonstrated alterations in the composition of the gut microbiomes of pigs infected with *S*. *enterica* either experimentally or naturally. In addition, we showed that the pig pathogen, *L*. *intracellularis*, was able to cause perturbations to the pig gut microbiome.

Using two separate tools for data analysis, PCoA and MetaStats, the composition of the gut microbiome in the cecum and colon was shown to be perturbed by challenge with either *S*. Typhimurium or *L*. *intracellularis* or both. PCoA was used to compare the overall microbiome compositions and structures while MetaStats was used to identify statistically significant differentially abundant bacteria. Based on PCoA, the compositions and structures of the colonic or cecal microbiomes of experimentally infected pigs were more similar to each other compared to the non-challenged control pigs, regardless of the challenge organisms. PCoA demonstrated a clustering of the microbiome compositions in infected animals when they were 7, 9, and 11 weeks of age, while the non-challenged controls segregated in different sectors of the PCoA plots. However, similar changes were not observed in the jejunal or ileal tissues. Since *S*. Typhimurium preferentially colonizes the cecum and colon, it isn’t surprising that dysbiosis of the jejunum and ileum did not occur in the *S*. Typhimurium infected pigs.

Using MetaStats, perturbations of the microbiome in the cecum and colon of the experimentally infected pigs were identified at the phylum and genus levels. For example (see Table 1), at 7 weeks of age (1 week post challenge with *S*. Typhimurium and 2 weeks post challenge with *L*. *intracellularis*) overall levels in the phyla Bacteroidetes and Actinobacteria were reduced in the dual challenged or *L*. *intracellularis* challenged pigs, respectively. Using a mouse model, Deatherage et al. showed similar reductions in bacteria in the phylum Bacteroidetes in fecal samples after challenge with *S*. *enterica* (12). However, while they also saw reductions in Firmicutes, our study in pigs did not show a similar decrease. At 9 weeks of age there were reductions in Spirochaetes for all challenged pigs and reductions in Fibrobacteres for pigs challenged with *S*. Typhimurium. At 11 weeks of age, there were reductions in Verrucomicrobia in pigs challenged with *S*. Typhimurium while pigs challenged with *L*. *intracellularis* exhibited reductions in Proteobacteria. In addition, numerous genera were identified that became more abundant after the challenges or less abundant when compared to the non-challenged control pigs (Table 2). These data taken together demonstrate that both pathogens are able to perturb the cecal and colonic microbiomes of pigs. *L*. *intracellularis* is known to mainly colonize the ileum of pigs and causes localized tissue damage [29] although *L*. *intracellularis* also can colonize anterior portions of the colon [30]. Based on the data collected in this experimental protocol it was not possible to determine if the *L*. *intracellularis* induced microbiome changes in the cecum and colon were a direct result of this pathogen or due to effects that occurred in the ileum that resulted in indirect effects in the cecum and colon. However, since *L*. *intracellularis* was found in the cecum and colon samples of challenged pigs when the pigs were 9 and 11 weeks of age, direct interactions between these two microbes is possible.

In addition to changes in the cecal and colonic microbiomes, the experimental approach also allowed us to visualize differences in the microbial compositions of the microbiomes of the jejunum and ileum compared to the cecum and colon. While there were similarities in which bacteria were present in each GI location, their relative abundances were quite different. For example, Proteobacteria were relatively minor components of the cecum and colon but were more abundant in the jejunum and ileum (particularly the ileum). In addition, the data presented demonstrated the phenomenon of bacterial succession. Over time, the relative composition of all tissue sites changed as the microbiome progressed to a climax community composition [31].

This mode of visualization did show some large variations of microbiome compositions after challenge with *S*. *enterica* serovar Typhimurium. In the jejunum and ileum of challenged pigs at 7 weeks of age (1 week post challenge with *S*. *enterica*) there was an increase in *Prevotella* and a decrease in *Streptococci* (Fig 4). By 9 weeks of age, the levels of *Prevotella* in the jejunum and ileum were reduced to similar to that seen in uninfected controls, but the levels of *Streptococci* remained high relative to other members of the microbiome.

Taken together, the interpretation of the data from the experimental challenge experiments was that pathogens do perturb the gut microbiomes of pigs. However, to determine if this was a phenomenon restricted to experimentally challenged pigs we also examined the microbiomes of pigs naturally infected with *S*. *enterica* by using commercially raised pigs. The herd used contained pigs that shed *S*. *enterica* (serovars Manhattan and Typhimurium) at low levels. By measuring shedding over time, we were able to segregate pigs into groups that never shed *S*. *enterica* at any of the sampling times, those that shed *S*. *enterica* one or two times, and pigs that shed *S*. *enterica* three or more times. Pigs in the highest shedding group were shown to have similar microbiome compositions and structures using PCoA but were different than pigs that were not shedding *S*. *enterica* or those that shed *S*. *enterica* only one or two times. It should be noted, that the classification of these pigs into shedding categories was based on the five discrete sampling times and it was possible that if other times had been selected for sampling that other pigs would been have found to be shedders. Nonetheless, there was a correlation between high shedders and their microbiome compositions.

To investigate the compositions of the naturally infected pigs further, we used MetaStats to identify differentially abundant genera (see S3 Table). The microbiomes of the pigs that were shedding at the time of sampling were compared to the non-shedding pigs. Several observations were of note. Firstly, there was an overall increase in the number of differentially abundant genera over time. Secondly, pigs actively shedding *S*. *enterica* at 22 weeks of age had elevated levels of *Akkermansia* although this was not the case at earlier times of sampling. This might be significant because *Akkermansia* is known to enhance gut inflammation and in doing so may enhance infections caused by *S*. *enterica* [32]. Given that pigs that are 22 weeks of age are approaching market weight, any factors that influence *S*. *enterica* infections might also increase the risk of cross contamination at the time of transport to slaughter plants. However, it should be noted that at 22 weeks of age, only one pig was shedding *S*. *enterica* and thus, this might not apply to other animals shedding *Akkermansia* at the same age. It also was observed that the relative level of *Campylobacter* was reduced at three of the five sampling times. This result could suggest an incompatibility between carriage of *S*. *enterica* and *Campylobacter*.

Thirdly, there were commonalities between the differentially abundant bacteria found in the pigs experimentally challenged with *S*. *enterica* and those that were naturally infected. The list of microbes differentially present in the colon of pigs at any of the three experimental times to those differentially present in naturally infected pig feces at 10 weeks of age were compared. We picked those time points because they were the most similar times of sampling in both protocols. Note, that the comparison is between colonic tissues versus feces. A total of nine genera were in common between these two groups of pigs: *Barnesiella*, *Pseudobutyrivibrio*, *Prevotella*, *Lactobacillus*, *Anaerobacter*, *Roseburia*, *Fastidiosipila*, *Campylobacter*, and *Succinivibrio*. *Barnesiella* and *Pseudobutyrivibrio* were present in increased levels and the other genera were present at reduced levels. Since the direction of abundance was the same between experimentally infected pigs and naturally infected pigs, it is likely that these represent true effects of being infected with *S*. *enterica* and further suggested that this pathogen is able to direct changes in the overall composition of the gut microbiome.

## Conclusions

In summary, the data presented here demonstrated that the pathogen *S*. *enterica* can cause disruptions in the gut microbiomes of pigs whether experimentally introduced into pigs or through natural infections. A similar conclusion was drawn from other studies that used chickens [13] and mice [12]. The major changes in the gut microbiome occurred in the lower intestinal tract: the cecum, colon, and feces. However, changes could be detected in the jejunum and ileum of these pigs. There was a correlation between the changes found between experimentally challenged pigs and naturally infected commercial pigs. While not unexpected, the data from the experimentally infected pigs also showed that there was a shift in the composition of the gut microbiome over time (in non-infected pigs) and that there were differences in the microbiome compositions based on anatomic location in the intestinal tract.

## Supporting Information

S1 Fig Genus level microbiome composition at 5, 7, 9, and 11 weeks of age in the jejunum, ileum, cecum, and colon of control pigs. Each sample is expressed as the mean for the group.
(PDF)

S2 Fig Genus level microbiome composition at 5, 7, 9, and 11 weeks of age in the jejunum, ileum, cecum, and colon of pigs challenged with *S*. *enterica* serovar Typhimurium strain 798. Each sample is expressed as the mean for the group.
(PDF)

S3 Fig Genus level microbiome composition at 5, 7, 9, and 11 weeks of age in the jejunum, ileum, cecum, and colon of pigs challenged with *L*. *intracellularis* strain PHE/MN1-00. Each sample is expressed as the mean for the group.
(PDF)

S4 Fig Genus level microbiome composition at 5, 7, 9, and 11 weeks of age in the jejunum, ileum, cecum, and colon of pigs challenged with *S*. *enterica* serovar Typhimurium strain 798 and *L*. *intracellularis* strain PHE/MN1-00. Each sample is expressed as the mean for the group.
(PDF)

S1 Table Bacterial genera and relative numbers in tissue samples from pigs challenged with Salmonella enterica, Lawsonia intracellularis, or both at various times post challenge. (XLSX)

S2 Table Differentially abundant species in pig colons that are statistically unique. (DOCX)

S3 Table Differentially abundant genera between *Salmonella* shedders and non-shedders at each time point. (DOCX)
