# Supplementary figures and images for: Changes in the Porcine Intestinal Microbiome in Response to Infection with Salmonella enterica and Lawsonia intracellularis
Source: PLoS One. 2015 Oct 13;10(10):e0139106. doi: 10.1371/journal.pone.0139106 (PMC4604083; doi:10.1371/journal.pone.0139106)

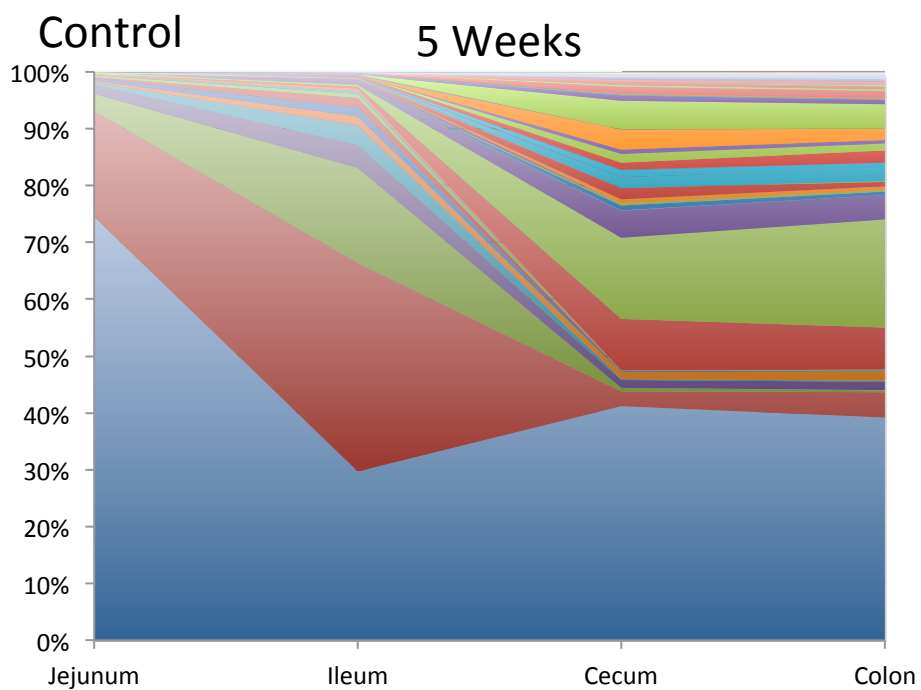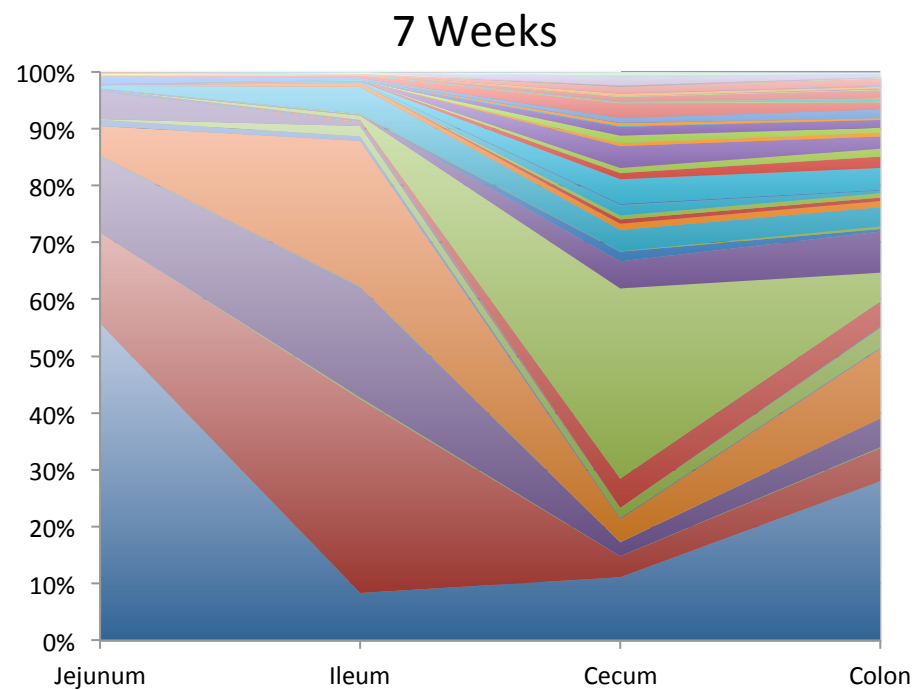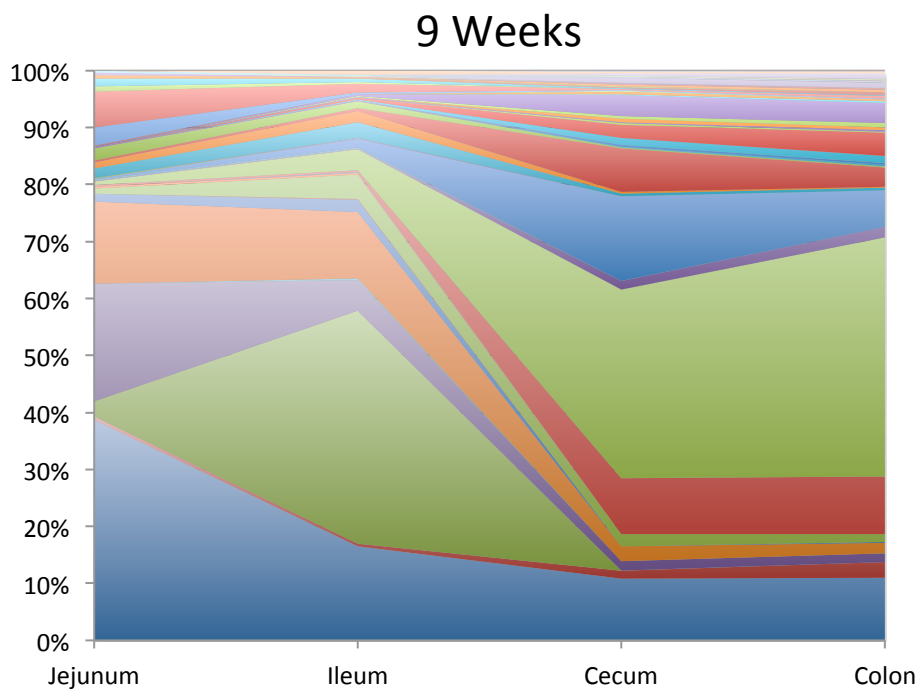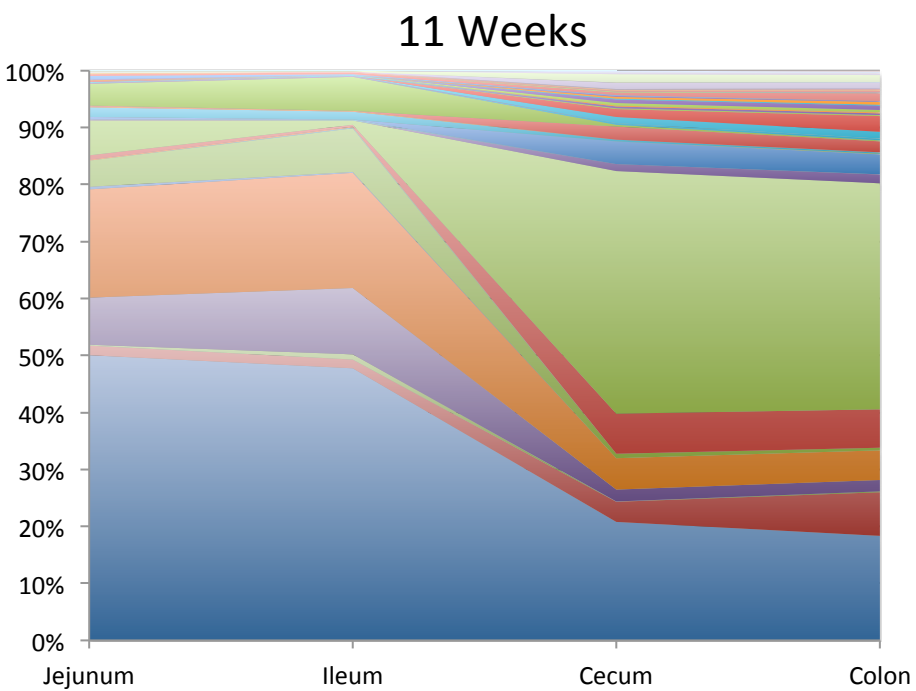

Supplement: S1 Fig — Each sample is expressed as the mean for the group. (PDF) [file pone.0139106.s001.pdf]

## Salmonella

5 Weeks

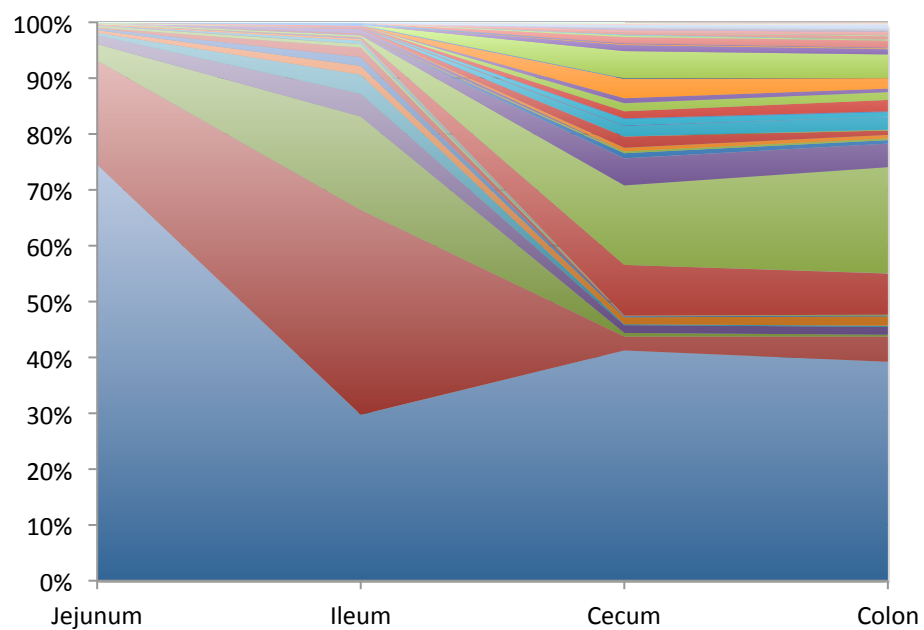

7 Weeks

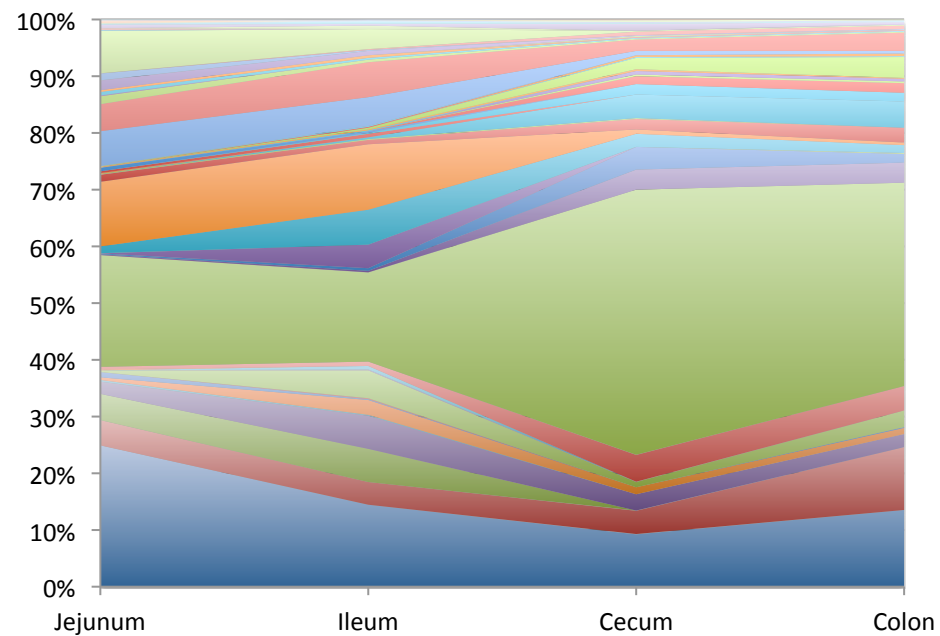

9 Weeks

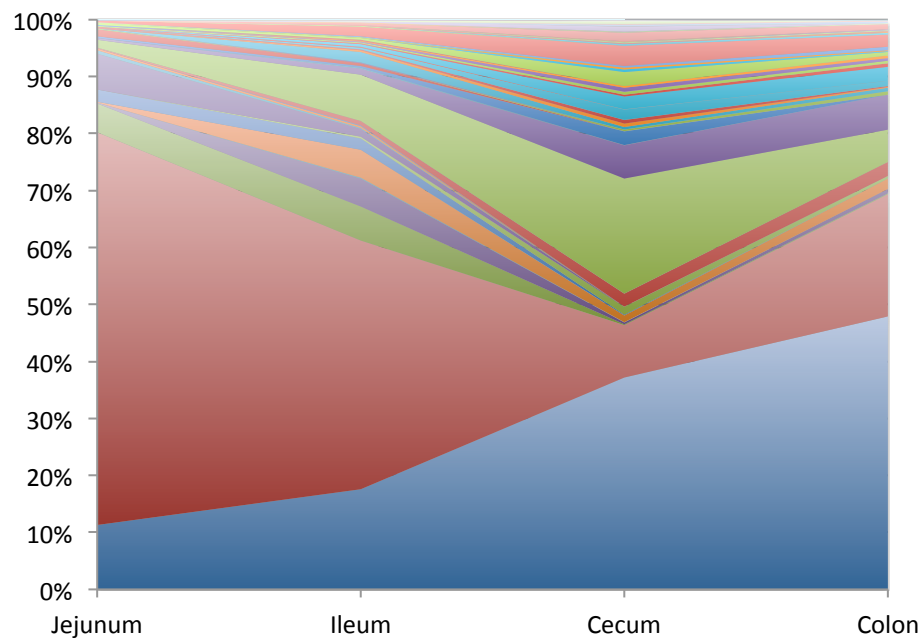

11 Weeks

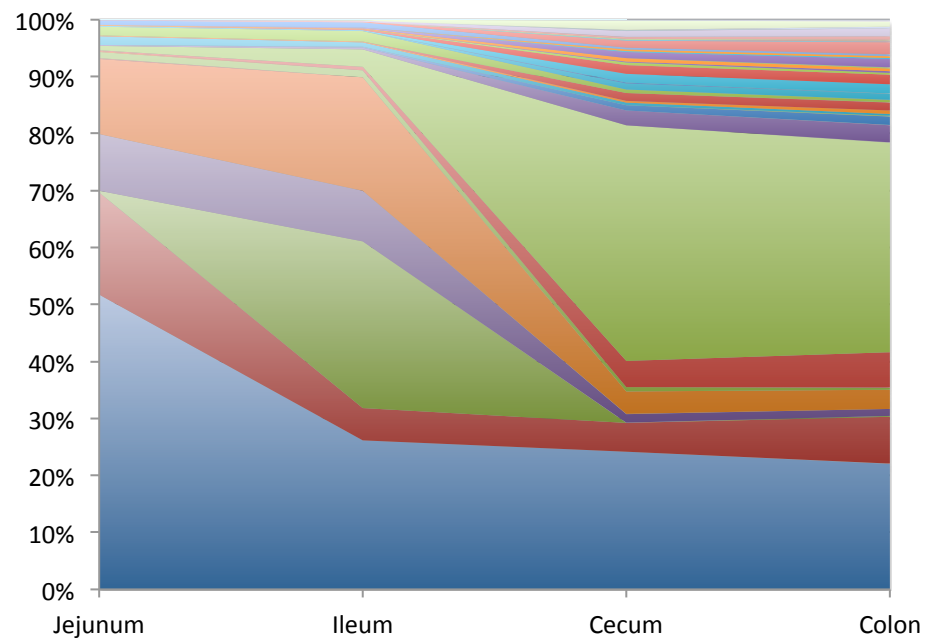

Supplement: S2 Fig — Each sample is expressed as the mean for the group. (PDF) [file pone.0139106.s002.pdf]

## Lawsonia

5 Weeks

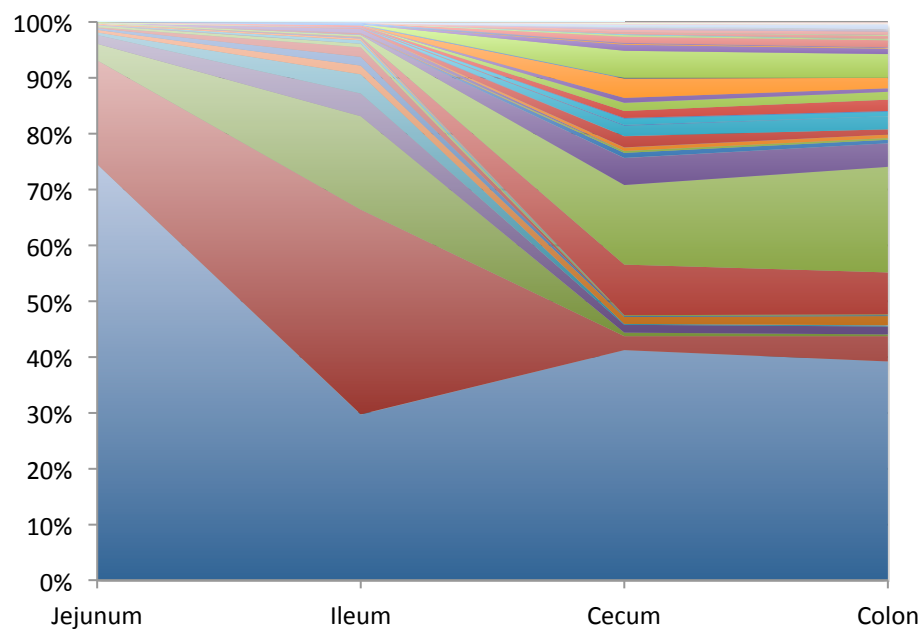

7 Weeks

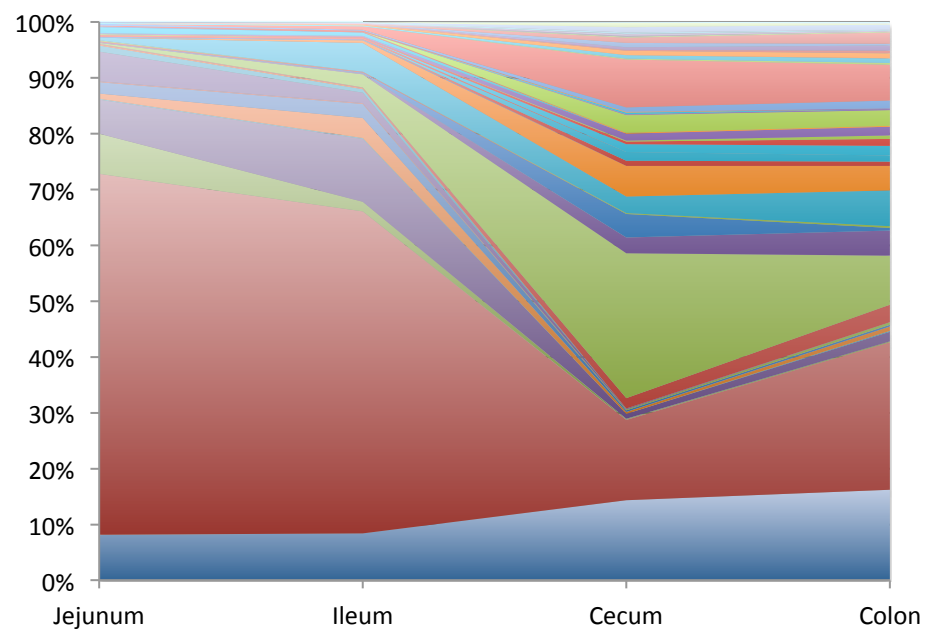

9 Weeks

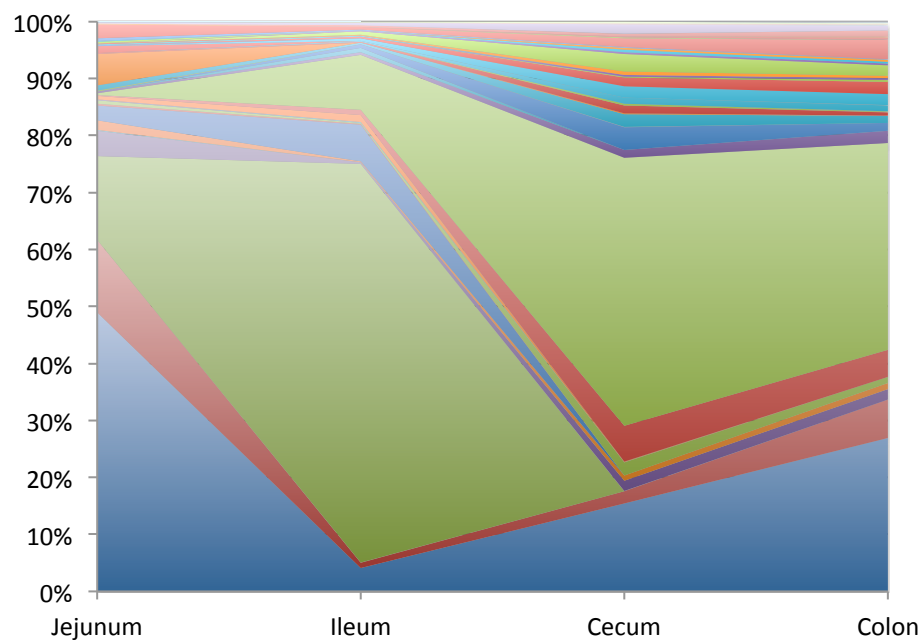

11 Weeks

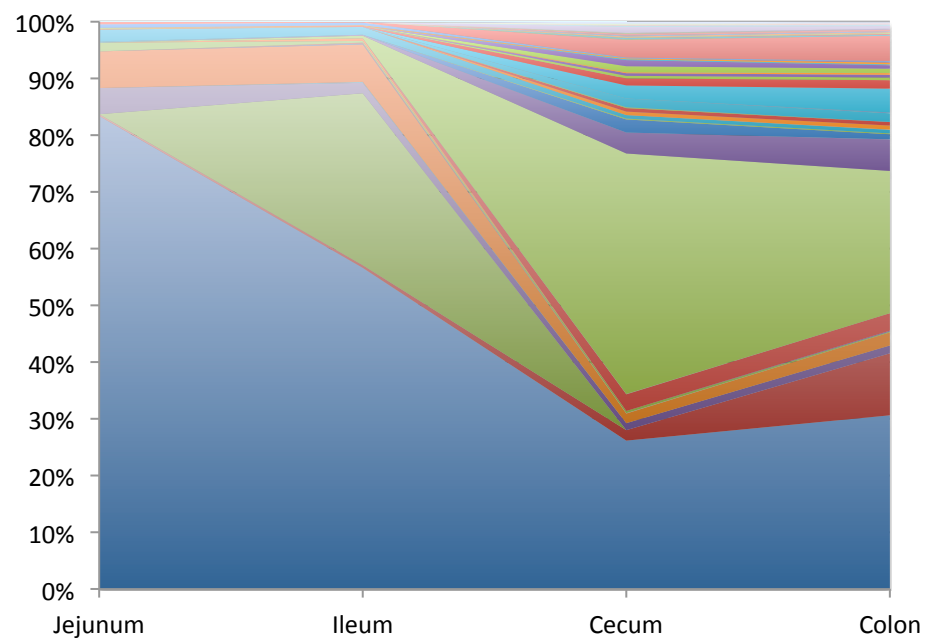

Supplement: S3 Fig — Each sample is expressed as the mean for the group. (PDF) [file pone.0139106.s003.pdf]

Salmonella and Lawsonia  
5 Weeks

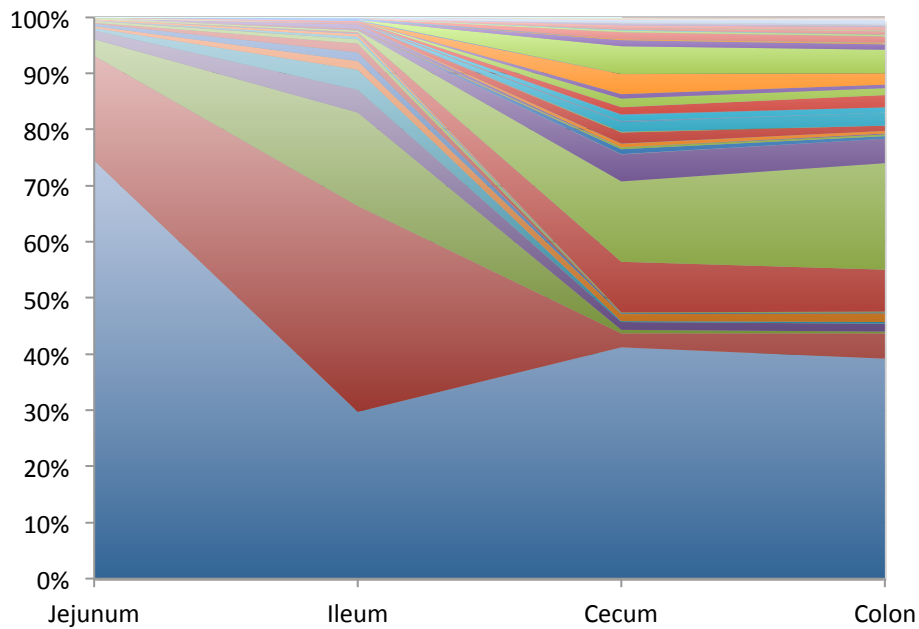

7 Weeks

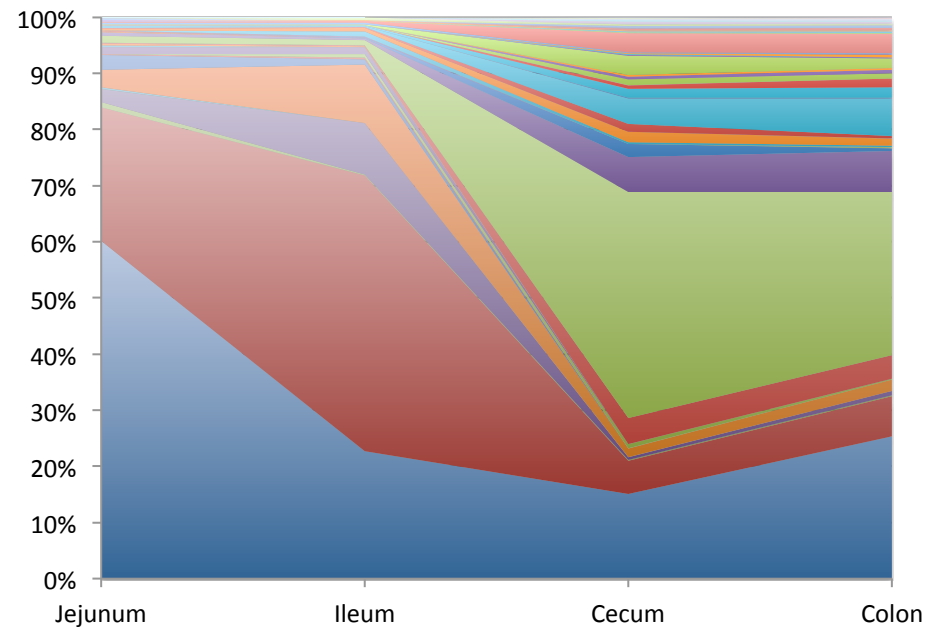

9 Weeks

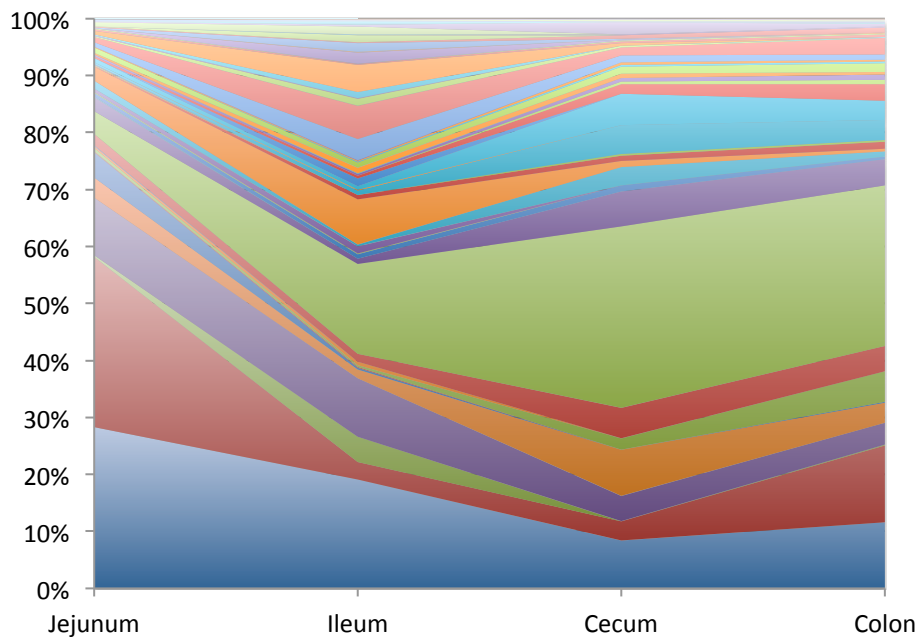

11 Weeks

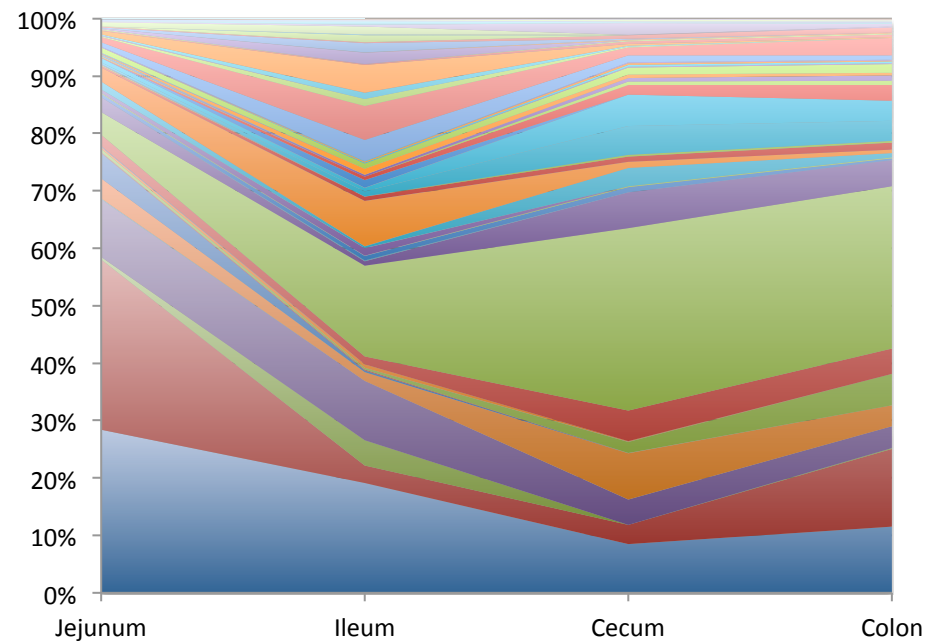

Supplement: S4 Fig — Each sample is expressed as the mean for the group. (PDF) [file pone.0139106.s004.pdf]
