# Supplementary material for: Changes in the Porcine Intestinal Microbiome in Response to Infection with Salmonella enterica and Lawsonia intracellularis
Source: PLoS One. 2015 Oct 13;10(10):e0139106. doi: 10.1371/journal.pone.0139106 (PMC4604083; doi:10.1371/journal.pone.0139106)
Supplement: S2 Table — (DOCX) [file pone.0139106.s006.docx]

S2 Table. Differentially abundant species in pig colons that are statistically unique

(p<0.05).

| 7 weeks | *Salmonella* | p-value | *Lawsonia* | p-value | Salmonella /Lawsonia | p-value |
| --- | --- | --- | --- | --- | --- | --- |
| Control^1^ | *Acetivibrio* ⭫  *Anaerobacter* ⭫  *Corynebacterium* ⭫  *Fastidiosipila* ⭫  *Hydrogenoanaerobacterium* ⭫  *Lactobacillus* ⭭  *Pediococcus* ⭫  *Roseburia* ⭫  *Sporacetigenium* ⭫  *Streptococcus* ⭭ | 6.4e-6  0.006  1.2e-5  0.0005  1.2e-5  0.014  5.1e-5  0.0003  3.6e-8  0.0007 | *Anaerobacter* ⭫  *Atopobium* ⭭  *Dorea* ⭫  *Fastidiosipila* ⭫  *Lachnobacterium* ⭫  *Lactobacillus* ⭫  *Spirochaeta* ⭫  *Sporacetigenium* ⭫  T*M7_genera_incertae_sedis* ⭫  *Turicibacter* ⭭ | 1.0e-5  0.0004  0.0001  0.0001  4.4e-5  0.013  7.1e-5  0.003  7.1e-5  0.0001 | *Anaerobacter* ⭫  *Barnesiella* ⭫  *Catenibacterium* ⭭  *Pediococcus* ⭫  *Prevotella* ⭭  *Pseudobutyrivibrio* ⭭  *Sporacetigenium* ⭫  *Turicibacter* ⭫  *Xylanibacter* ⭭ | 0.005  0.0004  0.0007  5.1e-5  4.7e-6  0.0006  0.002  2.0e-5  0.0001 |
| *Salmonella*^2^ |  |  | *Anaerofilum* ⭫  *Anaerovibrio* ⭭  *Atopobium* ⭭  *Erysipelothrix* ⭭  *Hydrogenoanaerobacterium* ⭫  *Lactobacillus* ⭫  *Parasporobacterium* ⭭  *Turicibacter* ⭭ | 0.0001  0.0006  0.0004  4.0e-6  1.2e-5  0.013  2.8e-5  3.3e-6 | *Hydrogenoanaero-*  *bacterium* ⭫  *Lactobacillus* ⭫  *Prevotella* ⭭  *Treponema* ⭭  *Xylanibacter* ⭭ | 1.2e-6  1.8e-5  0.001  0.0002  0.0001 |
| *Lawsonia*^3^ |  |  |  |  | *Atopobium* ⭫  *Bifidobacterium* ⭫  *Prevotella* ⭭  *Turicibacter* ⭫ | 7.8e-5  0.006  0.0001  8.7e-10 |
| 9 weeks |  |  |  |  |  |  |
| Control^1^ | *Acidaminococcus* ⭭  *Coprococcus* ⭫  *Dialister* ⭭  *Faecalibacterium* ⭭  *Mitsuokella* ⭭  *Oribacterium* ⭫  *Oscillibacter* ⭫  *Pseudobutyrivibrio* ⭭  *Shigella* ⭭  *Treponema* ⭫ | 2.3e-5  0.001  6.8e-5  1.6e-7  4.3e-5  2.4e-6  0.0009  0.004  2.6e-5  0.004 | *Coprobacillus* ⭫  *Oribacterium* ⭫  *Roseburia* ⭫ | 9.4e-6  1.9e-5  4.0e-8 | *Anaerobacter* ⭭  *Bifidobacterium* ⭭  *Coprobacillus* ⭫  *Faecalibacterium* ⭭  *Megasphaer* ⭭  *Oribacterium* ⭫  *Roseburia* ⭫  *Subdoligranulum* ⭭  *Treponema* ⭫  *Turicibacter* ⭫ | 0.001  0.0007  2.8e-5  8.2e-8  1.2e-5  0.001  0.001  0.001  0.0002  0.0005 |
| *Salmonella*^2^ |  |  | *Anaerofilum* ⭭  *Butyrivibrio* ⭫  *Catenibacterium* ⭫  *Fibrobacter* ⭭  *Hespellia* ⭭  *Lachnobacterium* ⭭  *Roseateles* ⭫  *Roseburia* ⭫  *Sporobacterium* ⭭ | 0.0003  0.0001  0.0002  1.5e-5  5.3e-5  0.0001  4.2e-5  0.002  6.4e-5 | *Anaeroplasma* ⭫  *Anaerosporobacter* ⭫  *Eubacterium* ⭭  *Fastidiosipila* ⭭  *Hespellia* ⭭  *Parasporobacterium* ⭭  *Peptococcus* ⭫  *Treponema* ⭭ | 2.8e-5  4.2e-6  8.5e-5  0.0005  2.8e-6  3.13-5  4.2e-5  0.0002 |
| *Lawsonia*^3^ |  |  |  |  | *Anaerobacter* ⭭  *Bifidobacterium* ⭭  *Erysipelothrix* ⭭  *Eubacterium* ⭭  *Faecalibacterium* ⭭  *Fibrobacter* ⭫  *Lachnobacterium* ⭫  *Paraprevotella* ⭭ | 2.2e-6  0.0007  0.0006  3.3e-5  0.0002  0.0001  5.5e-5  0.0001 |
| 11 weeks |  |  |  |  |  |  |
| Control^1^ | *Adlercreutzia* ⭫  *Akkermansia* ⭫  *Anaerovibrio* ⭫  *Campylobacter* ⭫  *Lebetimonas* ⭭  *Mogibacterium* ⭫  *Moryella* ⭭  *Paraprevotella* ⭫  *Selenomonas* ⭭ | 1.8e-8  0.001  6.4e-5  1.2e-5  3.4e-5  7.7e-6  7.1e-6  0.0002  7.2e-5 | *Barnesiella* ⭭  *Blautia* ⭭  *Coprococcus* ⭫  *Corynebacterium* ⭫  *Helicobacter* ⭫  *Hespellia* ⭫  *Paraprevotella* ⭫  *Succinivibrio* ⭫ | 6.1e-5  0.001  0.002  7.7e-6  7.7e-6  4.1e-6  0.0005  9.8e-8 | *Adlercreutzia* ⭫  *Akkermansia* ⭫  *Paraprevotella* ⭫  *Parasporobacterium* ⭫  *Sarcina* ⭫  *Streptococcus* ⭫  *Subdoligranulum* ⭭ | 9.7e-6  0.0001  0.0005  0.0002  0.0001  0.0002  0.0004 |
| *Salmonella*^2^ |  |  | *Alkaliphilus* ⭫  *Corynebacterium* ⭫  *Hallella* ⭫  *Moryella* ⭫  *Roseburia* ⭫  *Selenomonas* ⭫  *Slackia* ⭫ | 8.8e-6  1.0e-5  7.1e-7  7.13-6  5.4e-9  1.0e-8  3.5e-6 | *Moryella* ⭫  *Slackia* ⭫  *Sporobacter* ⭭  *Streptococcus* ⭫ | 7.1e-6  3.5e-6  3.4e-13  1.3e-7 |
| *Lawsonia*^3^ |  |  |  |  | *Blautia* ⭫  *Coprococcus* ⭭  *Parabacteroides* ⭭  *Sporobacter* ⭭ | 0.002  2.5e-6  0.0001  3.4e-13 |

* Statistically significant differences in microbiome composition based on an analysis using Metastats. Arrows indicate whether the difference was greater than (⭫) or less than (⭭ control pigs.

^1^ All samples across this row compared to control

^2^ All samples across this row compared to *Salmonella* challenge

^3^ All samples across this row compared to *Lawsonia* challenge
