# Supplementary material for: Changes in the Porcine Intestinal Microbiome in Response to Infection with Salmonella enterica and Lawsonia intracellularis
Source: PLoS One. 2015 Oct 13;10(10):e0139106. doi: 10.1371/journal.pone.0139106 (PMC4604083; doi:10.1371/journal.pone.0139106)
Supplement: S3 Table — (DOCX) [file pone.0139106.s007.docx]

S4 Table. Differentially abundant genera between *Salmonella* shedders and non-shedders at each time point

| 10 weeks |  | 13 weeks |  | 16 weeks |  |  |  |  |  |
| --- | --- | --- | --- | --- | --- | --- | --- | --- | --- |
| Genus | P value | Genus | P value | Genus | P value |  |  |  |  |
| Acidaminococcus 🡹 | 1.32E-04 | Adlercreutzia 🡹 | 2.83E-05 | Acetivibrio 🡻 | 2.66E-06 |  |  |  |  |
| Allisonella 🡹 | 1.17E-06 | Allobaculum 🡻 | 8.29E-12 | Allobaculum 🡻 | 1.59E-05 |  |  |  |  |
| Anaerobacter 🡹 | 1.97E-04 | Anaerobacter 🡹 | 9.63E-41 | Anaerococcus 🡹 | 7.44E-09 |  |  |  |  |
| Anaerofilum 🡹 | 3.10E-12 | Anaerovibrio 🡹 | 1.77E-20 | Anaerofilum 🡹 | 0.003572613 |  |  |  |  |
| Bacteroides 🡹 | 1.07E-20 | Bifidobacterium 🡻 | 2.08E-07 | Anaerosporobacter 🡻 | 0.000244058 |  |  |  |  |
| Barnesiella 🡹 | 3.40E-36 | Butyricicoccus 🡹 | 3.32E-21 | Anaerovibrio 🡹 | 5.89E-05 |  |  |  |  |
| Bifidobacterium 🡹 | 2.60E-08 | Catenibacterium 🡻 | 1.66E-13 | Bacteroides 🡻 | 7.67E-24 |  |  |  |  |
| Campylobacter 🡹 | 1.30E-05 | Coprococcus 🡻 | 6.96E-06 | Barnesiella 🡹 | 2.82E-11 |  |  |  |  |
| Dialister 🡻 | 3.19E-22 | Dialister 🡻 | 2.51E-06 | Bifidobacterium 🡻 | 4.65E-19 |  |  |  |  |
| Dorea 🡹 | 5.98E-06 | Dorea 🡹 | 2.32E-06 | Blautia 🡹 | 2.02E-113 |  |  |  |  |
| Enterococcus 🡹 | 1.40E-06 | Erysipelothrix 🡹 | 4.92E-06 | Butyricicoccus 🡹 | 3.76E-52 |  |  |  |  |
| Erysipelothrix 🡹 | 4.24E-09 | Ethanoligenens 🡹 | 2.97E-08 | Catenibacterium 🡹 | 1.94E-09 |  |  |  |  |
| Escherichia/Shigella 🡹 | 8.50E-13 | Faecalibacterium 🡹 | 0.003030158 | Desulfovibrio 🡻 | 8.21E-21 |  |  |  |  |
| Fastidiosipila 🡹 | 3.76E-06 | Fastidiosipila 🡻 | 2.77E-05 | Dialister 🡹 | 1.49E-83 |  |  |  |  |
| Hallella 🡹 | 4.43E-69 | Geopsychrobacter 🡻 | 2.03E-08 | Eubacterium 🡻 | 5.83E-09 |  |  |  |  |
| Lactobacillus 🡻 | 8.68E-64 | Hydrogenoanaerobacterium 🡻 | 0.000976317 | Faecalibacterium 🡹 | 9.84E-117 |  |  |  |  |
| Megasphaera 🡹 | 4.15E-04 | Lactobacillus 🡹 | 3.39E-137 | Fastidiosipila 🡻 | 6.62E-20 |  |  |  |  |
| Oscillibacter 🡹 | 5.27E-38 | Megasphaera 🡹 | 3.74E-09 | Fibrobacter 🡹 | 0.001488944 |  |  |  |  |
| Prevotella 🡻 | 1.46E-208 | Prevotella 🡻 | 2.80E-08 | Hydrogenoanaerobacterium 🡻 | 0.004423505 |  |  |  |  |
| Pseudobutyrivibrio 🡹 | 1.71E-62 | Pseudobutyrivibrio 🡻 | 3.32E-138 | Megasphaera 🡹 | 7.11E-230 |  |  |  |  |
| Roseburia 🡹 | 1.00E-20 | Roseburia 🡹 | 2.09E-06 | Mitsuokella 🡻 | 0.00463386 |  |  |  |  |
| Sharpea 🡹 | 8.33E-06 | Selenomonas 🡻 | 1.12E-12 | Oscillibacter 🡻 | 4.99E-37 |  |  |  |  |
| Sporobacter 🡹 | 1.95E-03 | Spirochaeta 🡻 | 1.49E-08 | Peptococcus 🡻 | 0.003954287 |  |  |  |  |
| Succinivibrio 🡹 | 4.05E-07 | Sporacetigenium 🡹 | 0.000251638 | Pseudobutyrivibrio 🡹 | 4.92E-05 |  |  |  |  |
| Treponema 🡹 | 1.82E-08 | Succinivibrio 🡹 | 5.06E-18 | Ruminococcus 🡻 | 4.04E-07 |  |  |  |  |
|  |  | Sutterella 🡹 | 9.09E-25 | Sarcina 🡹 | 0.009036205 |  |  |  |  |
|  |  | Turicibacter 🡹 | 1.52E-05 | Spirochaeta 🡻 | 5.07E-05 |  |  |  |  |
|  |  | Veillonella 🡹 | 1.43E-10 | Sporacetigenium 🡹 | 4.50E-05 |  |  |  |  |
|  |  | Xylanibacter 🡹 | 0.000488134 | Subdoligranulum 🡹 | 0.000170081 |  |  |  |  |
|  |  |  | 0.0012784 | Succinivibrio 🡹 | 0.000417871 |  |  |  |  |
|  |  |  |  | Sutterella 🡹 | 8.09E-13 |  |  |  |  |
|  |  |  |  | Tannerella 🡻 | 6.71E-05 |  |  |  |  |
|  |  |  |  | Treponema 🡻 | 9.25E-43 |  |  |  |  |
|  |  |  |  | Turicibacter 🡻 | 2.81E-10 |  |  |  |  |
|  |  |  |  |  |  |  |  |  |  |
|  |  |  |  |  |  |  |  |  |  |
|  |  |  |  |  |  |  |  |  |  |
|  |  |  |  |  |  |  |  |  |  |
|  |  |  |  |  |  |  |  |  |  |
|  |  |  |  |  |  |  |  |  |  |
|  |  |  |  |  |  |  |  |  |  |
| 19 weeks |  | 22 weeks |  |  |  |  |  |  |  |
| Genus | P value | Genus | P value |  |  |  |  |  |  |
| Anaerobacter 🡹 | 2.84E-59 | Acetanaerobacterium 🡹 | 6.69E-16 |  |  |  |  |  |  |
| Anaerofilum 🡹 | 8.65E-06 | Acetivibrio 🡻 | 1.94E-09 |  |  |  |  |  |  |
| Anaeroplasma 🡻 | 0.000244013 | Acidaminococcus 🡹 | 0.003905878 |  |  |  |  |  |  |
| Anaerovibrio 🡻 | 0.000312937 | Acinetobacter 🡻 | 0.007811922 |  |  |  |  |  |  |
| Atopobium 🡹 | 0.001512296 | Adlercreutzia 🡹 | 2.07E-05 |  |  |  |  |  |  |
| Bacteroides 🡻 | 1.03E-05 | Aerococcus 🡹 | 4.87E-06 |  |  |  |  |  |  |
| Barnesiella 🡹 | 9.71E-24 | Akkermansia 🡻 | 2.67E-06 |  |  |  |  |  |  |
| Blautia 🡹 | 1.23E-14 | Allisonella 🡹 | 7.22E-07 |  |  |  |  |  |  |
| Campylobacter 🡻 | 0.000121996 | Allobaculum 🡹 | 3.69E-05 |  |  |  |  |  |  |
| Coprococcus 🡻 | 0.000493979 | Alysiella 🡹 | 0.003905878 |  |  |  |  |  |  |
| Desulfovibrio 🡻 | 0.000315539 | Anaerobacter 🡹 | 8.19E-223 |  |  |  |  |  |  |
| Dialister 🡹 | 3.68E-52 | Anaerobiospirillum 🡹 | 0.003905878 |  |  |  |  |  |  |
| Faecalibacterium 🡹 | 1.12E-23 | Anaerofilum 🡹 | 1.10E-15 |  |  |  |  |  |  |
| Fastidiosipila 🡻 | 1.71E-18 | Anaerosporobacter 🡻 | 6.27E-50 |  |  |  |  |  |  |
| Hallella 🡻 | 7.83E-15 | Anaerotruncus 🡹 | 0.000820971 |  |  |  |  |  |  |
| Lactobacillus 🡹 | 1.86E-134 | Anaerovibrio 🡻 | 1.27E-18 |  |  |  |  |  |  |
| Megasphaera 🡹 | 5.87E-120 | Bacteroides 🡻 | 1.36E-28 |  |  |  |  |  |  |
| Mitsuokella 🡹 | 1.67E-14 | Barnesiella 🡻 | 7.14E-08 |  |  |  |  |  |  |
| Oscillibacter 🡻 | 2.58E-05 | Bifidobacterium 🡹 | 1.13E-53 |  |  |  |  |  |  |
| Parasporobacterium 🡻 | 9.99E-23 | Blautia 🡹 | 7.04E-266 |  |  |  |  |  |  |
| Prevotella 🡻 | 2.15E-15 | Butyricicoccus 🡹 | 7.05E-57 |  |  |  |  |  |  |
| Pseudobutyrivibrio 🡻 | 2.62E-57 | Butyrivibrio 🡹 | 8.33E-121 |  |  |  |  |  |  |
| Roseburia 🡻 | 4.19E-75 | Campylobacter 🡻 | 4.05E-07 |  |  |  |  |  |  |
| Spirochaeta 🡻 | 3.81E-06 | Catenibacterium 🡻 | 2.47E-06 |  |  |  |  |  |  |
| Sporacetigenium 🡹 | 2.69E-28 | Clostridium 🡹 | 4.24E-93 |  |  |  |  |  |  |
| Streptococcus 🡹 | 2.24E-205 | Coprococcus 🡻 | 0.00E+00 |  |  |  |  | Xylanibacter 🡻 | 1.61E-17 |
| Subdoligranulum 🡹 | 7.30E-08 | Corynebacterium 🡹 | 0.000402321 |  |  |  |  |  |  |
| Tannerella 🡻 | 5.83E-15 | Desulfovibrio 🡻 | 5.46E-20 |  |  |  |  |  |  |
| Treponema 🡻 | 1.31E-88 | Dialister 🡹 | 1.36E-171 |  |  |  |  |  |  |
| Xylanibacter 🡻 | 2.54E-18 | Dorea 🡹 | 4.15E-27 |  |  |  |  |  |  |
|  |  | Eggerthella 🡻 | 0.006984871 |  |  |  |  |  |  |
|  |  | Erysipelothrix 🡹 | 3.88E-56 |  |  |  |  |  |  |
|  |  | Escherichia/Shigella 🡻 | 2.50E-10 |  |  |  |  |  |  |
|  |  | Ethanoligenens 🡻 | 5.48E-13 |  |  |  |  |  |  |
|  |  | Eubacterium 🡻 | 3.22E-05 |  |  |  |  |  |  |
|  |  | Faecalibacterium 🡹 | 1.73E-81 |  |  |  |  |  |  |
|  |  | Fastidiosipila 🡻 | 7.70E-97 |  |  |  |  |  |  |
|  |  | Gordonibacter 🡹 | 2.98E-09 |  |  |  |  |  |  |

Statistically significant at p<0.05. Arrows represent an increase or decrease in each genera compared to pigs not shedding *S. enterica*
